# The Effect of Shift Working on Dietary Patterns of Healthcare Practitioners during the COVID-19 Pandemic: A Cross-Sectional Study

**DOI:** 10.3390/medicina60040627

**Published:** 2024-04-12

**Authors:** Athanasios Migdanis, Konstantinos Tsolis, Ioannis Migdanis, Agoritsa G. Kaltsa, Fotios A. Fytsilis, Athanasios Manouras, Odysseas Androutsos, Andreas Kapsoritakis

**Affiliations:** 1 Nutrition and Dietetics Department, University of Thessaly, Argonafton 1C, 42132 Trikala, Greece; johnymig@hotmail.com (I.M.); amanouras@uth.gr (A.M.); 2 Faculty of Medicine, University of Thessaly, Viopolis Mezourlo, 41110 Larissa, Greece; tsoliskostas@yahoo.gr (K.T.); kapsoritakis@uth.gr (A.K.); 3 Department of Gastroenterology, General University Hospital of Larissa, Viopolis Mezourlo, 41110 Larissa, Greecefotisf_thess@hotmail.com (F.A.F.); 4 Laboratory of Clinical Nutrition and Dietetics (CND-Lab), Department of Nutrition and Dietetics, University of Thessaly, Argonafton 1C, 42132 Trikala, Greece; oandroutsos@uth.gr

**Keywords:** nutritional patterns, healthcare professionals, shift working

## Abstract

*Background and Objectives:* Health professionals’ working schedules often do not allow them to follow a balanced way of life, and the nature of their work can affect their weight and eating habits. The aim of the present study is to shed some light on the impact of night shift working on the dietary practices of healthcare practitioners in Greece. *Materials and Methods:* This was a cross-sectional study. With the use of an administered questionnaire several parameters were assessed, including anthropometric characteristics, weight history, as well as nutritional habits of the same group of participants during night shifts and when at regular working hours. Moreover, nutritional beliefs of the subjects were recorded, as well as the level of adherence to the Mediterranean diet. *Results:* It was observed that in all food groups, including water, used to compare the nutritional patterns of the participants during night shifts and when working regular hours, statistical differences were noted (*p* = 0.001). Furthermore, the participants who reported a higher adherence to the Mediterranean diet and self-reported healthier nutritional habits, had a statistically lower weight compared to the participants that rarely followed the specific model or reported unhealthier eating routine (*p* = 0.010 and *p* = 0.015, respectively). *Conclusions:* We conclude that shift-working healthcare professionals are associated with disturbed eating behaviours leading to the high consumption of unhealthy food. The implementation of interventions that will concentrate on educating health workers on balanced nutrition and improving physical environment (i.e., food availability, healthier food choices) seems to be of great importance.

## 1. Introduction

Maintaining a healthy way of life and a balanced diet is crucial for healthcare workers to perform optimally, especially when shift working, where their resilience and well-being are put to the test. Health professionals’ working schedules often do not allow them to follow a balanced lifestyle, and the nature of their work can affect their weight, their eating habits, their sleep routines, their emotional state and their performance at work [1,2,3,4]. Studies have found that declining lifestyle factors, particularly increased body weight and alcohol intake, as well as decreased fruit and vegetable consumption in clinicians and shift workers in general, is associated with higher odds of the incidence of metabolic syndrome, cardiovascular disease, and other chronic conditions [5,6,7]. It has also been reported by previous studies that individuals with disrupted sleeping patterns and high levels of emotional stress such as shift-working healthcare professionals, might present an increased rate of eating disorders (i.e., anorexia nervosa, binge eating disorder, or orthorexia nervosa) [8,9,10].

The coronavirus disease 2019 (COVID-19) pandemic has posed unprecedented challenges to the healthcare sector worldwide, affecting both patient care and the well-being of healthcare professionals. In Greece, like many other countries, the pandemic has placed immense pressure on healthcare facilities, particularly in regional general hospitals. Amidst the uncertainties and demands of managing the pandemic, the health and well-being of healthcare workers have emerged as a critical concern. Apart from the immediate health risks they face on the frontlines, the pandemic has also disrupted their daily routines, work patterns, and lifestyle choices [11]. The pandemic’s impact on the well-being of healthcare workers has been profound [12]. Long hours, exhaustion, and psychological stress can potentially lead to changes in dietary behaviours and eating patterns. The disruption of daily routines, limited access to certain foods, and psychological factors related to the pandemic may influence food choices and dietary practices among healthcare workers [11,13].

Understanding the dietary habits of healthcare workers is vital for several reasons. Firstly, it provides valuable insights into the overall well-being and resilience of this critical workforce. Dietary behaviours can significantly impact physical and mental health, affecting the ability of healthcare professionals to cope with the challenges posed by the pandemic effectively [11,14]. Secondly, examining dietary habits can identify potential areas for intervention and support [15]. Health institutions and policymakers can use the findings to develop targeted programs that promote healthy eating habits and address nutritional deficiencies among healthcare workers. Additionally, such insights can help design strategies to alleviate stress and enhance overall well-being, ultimately improving their ability to deliver quality patient care.

The present scientific article aims to examine the dietary habits of healthcare workers employed in regional general hospitals in Greece during the COVID-19 pandemic. The study focuses on understanding the frequency of food and beverage consumption at various stages, including when healthcare workers work day hours and during their night shifts, particularly during periods of extended work hours and high-stress situations. Furthermore, this study seeks to contribute to the growing body of knowledge on the impact of shift work on the dietary habits of healthcare workers in regional general hospitals in Greece. By examining the frequency of food and beverage consumption and understanding the dietary patterns followed by this workforce, we aim to help build an evidence base that in the future will contribute by providing recommendations that will improve the overall health and well-being of healthcare professionals during and beyond a pandemic.

Although research in the existing literature has detected poor dietary practices and a general unhealthy lifestyle in healthcare workers, scientific data on the nutritional differences between day workers and night shift workers are still sparse and with some limitations. Existing studies mainly focus on female population and compare different groups of healthcare workers that either work regular day shifts or night hours [16,17,18,19]. The main aim of the present study is to shed some light on the impact of night shift working on the dietary practices of Greek healthcare practitioners (a very understudied population). A strength of this study, in contrast to other, is the observation of the same group of healthcare professionals at different time points (when working regular day hours and at night shifts). Other parameters including anthropometric characteristics and nutritional beliefs and knowledge of the subjects were also assessed.

## 2. Materials and Methods

This was a cross-sectional study. The study was initiated in September 2020 and completed in September 2021. The study was conducted among 418 male and female medical staff working at two regional public hospitals in Greece. With the use of an administered questionnaire, several parameters were assessed and recorded, including the anthropometric characteristics, level of education, recent medical, drug, and weight history, and nutritional habits of the participants during night shifts and when at normal working hours. The questionnaire was administered to all the medical staff employed at both hospitals based in the city of Ioannina, University General Hospital of Ioannina and General Hospital Ioanninon Chatzikosta, Greece. The questionnaire was administered both electronically and in printed form. A registered dietitian was available for contact in case of needed clarifications. Participants who had an acute or chronic condition (e.g., those with food allergies, inflammatory bowel disease, chronic renal failure, or pancreatitis, had undergone major surgery, had a recent infection, or were undergoing chemo/radiotherapy) that might have affected their recent nutritional status or intake were excluded from the study. Also, 33 people, 22 male and 11 female, refused to participate in the study.

To assess the dietary habits of the participants, the food frequency approach was used which asks respondents to report their usual frequency of consumption of each food or food group from a list of foods for a specific period [20]. It is a valid and accepted method/tool that can help assess/estimate the frequency of consumption and “habitual” intake of foods and beverages [20]. More specifically, the questionnaire used addressed the nutritional habits of the participants separating the main food groups into fruit, vegetables, legumes, red meat, poultry/fish, sweets and confectionery, alcohol, dairy, and lunch meat. The frequency of the consumption of the above reported food groups was assessed. Moreover, nutritional beliefs and knowledge of the subjects was recorded, as well as the level of adherence to the Mediterranean diet. Information regarding water consumption and differences in 24 h nutritional practices when working night shifts and when being on day working hours were also documented. To assess the level of consumption of the above food groups and the general nutritional habits of the participants, a 5-point Likert type scale was used, ranging from 0 = no consumption to 5 = daily consumption, respectively. Informed consent was obtained from all participants before entering the study. The questionnaire was first developed having an initial set of questions which were reformed after piloting the questionnaire once to 20 respondents. Piloting the questionnaire helped refine the wording and the layout in terms of comprehension and response difficulties. The study protocol was approved by University General Hospital of Ioannina and General Hospital Ioanninon Chatzikosta Ethics Committee (ethics approval code no. 12386/6-5-2019 and 4/11-4-2019), and adhered at all times to the Helsinki Declaration. The study was also approved by the Ethics Committee of the University of Thessaly (ethics approval code no. 02/12.2.2019).

### Statistical Analysis

Statistical analysis was conducted using Statistical Product and Service Solutions, version 26 (IBM SPSS Statistics for Windows, Armonk, NY, USA). The Kolmogorov–Smirnov test was carried out to determine the normality of the distribution of the examined variables. Continuous variables such as anthropometric characteristics are presented as means and ± standard deviations. To assess possible differences between 24 h dietary patterns when working regular hours (8.00 a.m.–4.00 p.m.) compared to night shift hours (8.00 a.m.–8.00 a.m.), cross tabulation analysis was used. To examine the possible relation between adherence to the Mediterranean diet and personal beliefs about eating habits and weight changes, one-way ANOVA was used. Statistical significance was maintained at *p* < 0.05.

## 3. Results

A total of 418 participants consented and were recruited for the study. A wide range of the participants was between the age of 36 and 45 years (32.3%) (68.7% males and 31.3% females). The mean BMI of the participants was slightly above the normal range 25.7 ± 15.5 kg/m^2^, which falls within the overweight category (Table 1).

**Figure 1:**
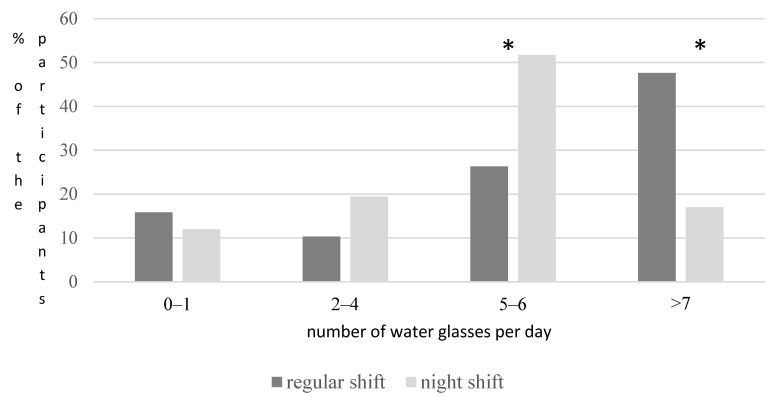
Comparison between water consumption when on regular working hours and on night shift hours. * Indicates significant differences in water consumption between the two groups (*p* < 0.05).

Regarding the nutritional habits of the participants, 24.6% reported they rarely, 39.2% reported they often, and 36.1% reported they always followed the Mediterranean diet. Moreover, it was observed that in all food groups used to compare the nutritional patterns of the participants during night shifts and when working normal hours, statistical differences were noted (*p* = 0.001) (Table 2). More specifically, participants significantly less frequently consumed fruit and vegetables during night shift hours compared to when working daytime hours (*p* = 0.001). On the other hand, the consumption of sweets and lunch meat was significantly higher during night shift hours compared to when working daytime hours (*p* = 0.001) (Table 2). As far as hydration is concerned, water consumption was found to be significantly lower when participants were working shift hours compared to regular daytime hours (*p* = 0.001) (Figure 1).

Furthermore, it was observed from the analysis conducted that the participants who reported a higher adherence to the Mediterranean diet had a statistically lower weight compared to the participants that rarely followed the specific model (*p* = 0.010) (Table 3). Lastly, according to the question where participants self-assessed/categorized their nutritional habits from ideal to not healthy, weight was again significantly lower for the subjects that had reported healthier eating habits compared to those that had reported not healthy eating habits (*p* = 0.015) (Table 4).

## 4. Discussion

The study’s findings indicate that medical staff working on shifts tends to maintain a body weight above the proposed cutoffs. These findings seem to be in agreement with similar studies that have assessed the anthropometric characteristics of such groups. In a study from Germany which was conducted on shift-working healthcare personnel, participants showed a tendency towards overweight presenting a mean BMI of 25.7 kg/m^2^ [16]. This study aimed to identify differences in lifestyle habits in individuals employed in healthcare in Germany and working on either a shift or a non-shift schedule [16]. In another study, which took place in the UK and in which the authors focused on dietary habits and anthropometric characteristics of hospital staff, the results showed that 64.5% of the clinical staff that participated were overweight or obese [21]. It has been suggested that employees in the healthcare industry are frequently facing different working patterns that might influence their health and both psychological and physical well-being [22]. Shift work poses difficulties not only because of the loss of actual sleep hours but also because it can affect other factors related to lifestyle, such as food intake, physical activity level and therefore metabolic patterns [22].

With regard to the nutritional patterns of the participants in the present study, it was observed that there was a significant difference in dietary intake when on shifts compared to when working non-shift schedules. According to the existing literature on the subject, shift workers often adopt imbalanced nutritional patterns, mainly relying on unhealthy foods high in energy and sugar and showing a low consumption of healthy snacks such as fruits and vegetables [17,23]. This can mainly be attributed to the lack of time availability during shift work, which seems to be a major barrier to consume healthier food. Since shift workers work irregular hours, their daily routine seems to be interrupted. Regular eating and exercise habits are often difficult to maintain [4]. The physical environment plays an important role in determining health behaviours and the hospital environment may have an impact on workers’ eating behaviours. Healthy food option availability often seems to be an important problem as there is limited access to healthy food and inadequate food storage and preparation areas in hospitals generally. In a study from Canada, the authors tried to examine the relationship between shift schedule, body mass index, and nutritional habits among a large sample of nurses (*n* = 9541) [24]. Respondents were asked about facilities provided by their employer related to physical activity and places for staff to purchase healthy food. The results showed that the access to healthy food was limited overall and especially during shifts, with 41.2% of participants reporting healthy food not being available at all. In the present study, the results revealed that participants significantly less frequently consumed fruit and vegetables during shift hours compared to when working daytime hours. These results seem to be in agreement with similar studies that have tried to assess the same parameters. In an observational study that tried to compare health behaviours of internal medicine residents and medical students, the results showed that both groups frequently failed to achieve the recommended daily servings of fruits and vegetables, with residents consuming significantly fewer servings than medical students [17]. In another study, which aimed to record food intake in resident physicians, again the analysis yielded similar outcomes [18]. Food intake was determined through a self-administered food diary that was kept over the course of three nonsuccessive days. Poor diet was observed for both genders, including a low intake of vegetables and fruits and a high intake of sweets. Further literature on the subject examining workforce nutrition has also reported nurses consuming similarly low quantities of fruit and vegetables compared to other populations [25,26]. A study that tried to compare the dietary habits of nurse shift workers and nurse day workers revealed that shift workers consumed significantly lower amounts of fruit and vegetables. The study was conducted in Japan among 1179 day workers and 1579 rotating shift workers [18]. Explanations for the above findings seems to be fatigue from working long hours and irregular meal frequency, which may lead to unhealthy snacking behaviours [27].

In an effort to understand the nutritional behaviours/habits of such populations, the consumption of sweets/confectionery and lunch meat was also assessed in our study. The analysis of the data collected showed that the consumption of sweets and lunch meat was significantly higher during shift hours compared to when working daytime hours. It has been documented in the literature that night shift workers are less likely to have regular, full meals and often replace meals with unhealthy snacks and convenience food [19]. Ironically, in healthcare environments, junk food is more easily accessible and cheaper than healthy alternatives. Moreover, sweets and chocolates are often offered from patients or their relatives to medical staff as a form of gratitude for their services is a common social feature in many countries. Other studies on the subject seem to produce similar results. In a study from Japan which aimed to examine the influence of shift work on lifestyle and diet in female nurses and caregivers, it was seen that shift workers consumed more sweet beverages and snacks than day workers. In addition, shift workers reported irregular meal patterns often missing meals such us breakfast or lunch [28]. In a study where the authors attempted to evaluate the physical activity, energy expenditure and nutritional habits of healthcare personnel working in rotating shifts or regular hours observed that the shift working group had a significantly higher consumption of sugar [16]. In a systematic review on shift working and nutrition, the authors suggest that shift working can be an occupational hazard possibly due to an impairment of biological rhythms. The paper concludes that since people work irregular hours their daily routine is interrupted, leading to difficulties in maintaining balanced eating. Shifts were generally found to significantly influence the intake of starches and sweets [29].

As far as hydration is concerned, the results of the present study showed that water consumption was significantly lower when participants were working shift hours compared to regular daytime hours. Physicians and nurses are often unable to meet their fluid requirements during shifts, and this could affect their hydration status and hence decision making and cognitive performance [30,31,32]. There is lack of thorough research examining the relationship between hydration status and its effect on healthcare staff during shifts. In a prospective cross-sectional study that tried to assess the hydration status of emergency department physicians and nurses, the results showed that the majority of physicians and to a lesser extent nurses had decreased hydration status at the end of their shifts [33]. In a similar study from the UK, which tried to investigate the prevalence of dehydration at the start and end of shifts in nurses and doctors on-call, the authors observed that a significant proportion of nurses and doctors were dehydrated at the start and end of medical and surgical shifts [34].

Furthermore, in an effort to understand nutritional behaviours/habits of such a population group, the level of compliance of the participants to the Mediterranean diet model was assessed according to the participants’ self-report. The Mediterranean diet is a plant-based diet comprising high amounts of vegetables, fruit, cereal, nuts, and extra virgin olive oil, a moderate consumption of fish, poultry, and dairy products, and a low intake of processed red meat products and saturated fats [35]. The Mediterranean diet has been recognized as a balanced beneficial nutritional pattern that decreases the risks of a variety of human disorders and pathological states and promotes human health [36]. It was observed from the analysis conducted that the participants who reported a higher adherence to the Mediterranean diet had a statistically lower weight compared to the participants that rarely followed the specific model. Also, according to the question where participants self-assessed their nutritional habits from ideal to not healthy, weight was again significantly lower in the subjects that had reported healthier eating habits compared to those that had reported not healthy eating patterns. Shift work is associated with a higher frequency of chronic diseases such as insulin resistance, diabetes mellitus, dyslipidaemia, metabolic syndrome, cardiovascular disease, cancer, and gastrointestinal disorders [37,38,39,40]. A reduced amount and/or quality of sleep, physical inactivity, poor dietary practices, positive energy balance, and overweight and obesity appear to be the most important causative factors. In workplaces, strategies should be implemented to promote healthy eating focusing on nutritional counselling/education and behavioural change. Additionally, some programs should focus on changing the worksite environment in order to make healthy dietary choices easier. Limitations of the study include restricted sample size and a double-centre data collection which can potentially affect the results’ external validity, but our findings can add information/evidence to the existing literature and suggest directions for future research with larger sample sizes and a multicentre design. Lastly, the reliance on self-reported questionnaires can introduce recall bias and social desirability bias, affecting the accuracy of reported dietary habits. Also, with food frequency questionnaires, little detail is collected on other characteristics of the foods in terms of the way they are consumed, such us the methods of cooking or the combinations of foods in meals.

## 5. Conclusions

We conclude, according to the results of the study, that shift-working healthcare professionals are associated with increased body weight and disturbed eating behaviours leading to high consumption of unhealthy food. This situation was likely exacerbated following the COVID-19 pandemic pressure. Future studies should focus on assessing the body composition (e.g., body fat percentage) of such populations and quantifying the daily intake of a broad range of micronutrients. The implementation of interventions that concentrate on educating health workers on proper, balanced nutrition and improving the physical environment (i.e., food availability, healthier food choices) seems to be of great importance. Lasty, the creation of nutritional guidelines and practical recommendations that could help meet the requirements of such a special group of workers would contribute to the improvement of their general health status.

## Figures and Tables

**Table 1:** Demographics, anthropometric characteristics, and nutritional habits of the participants.

Variables	Number of Participants (%)/Mean ± SD
Gender	
Males	287 (68.7)
Females	131 (31.3)
Weight (kg)	80.4 ± 15.5
BMI (kg/m^2^)	25.7 ± 3.9
Age (years)	
22–35	124 (29.7)
36–45	135 (32.3)
46–55	82 (19.6)
56–67	77 (18.4)
Years at work	
<5	90 (21.5)
5–10	91 (21.8)
11–20	113 (27)
21–30	108 (25.8)
>31	16 (3.8)
Following Mediterranean diet	
rarely	103 (24.6)
often	164 (39.2)
always	151 (36.1)
Breakfast at hospital (on night shift)	
Never	160 (39)
Rarely	130 (31.7)
Often	65 (15.9)
Very often	43 (10.5)
Always	12 (2.9)
Snacks at hospital (on night shift)	
Never	110 (26.3)
Rarely	14 (3.3)
Often	139 (33.3)
Very often	148 (35.4)
Always	7 (1.7)
Food derived from (on night shift)	
Hospital’s restaurant	7 (2.3)
Hospital’s canteen	19 (6.2)
From home	126 (40.9)
Take away	156 (50.6)

BMI, body mass index; SD, standard deviation.

**Table 2:** Comparison in nutritional intake between when working regular hours and night shift hours.

Food Groups	Frequency	No of Participants (%) (Day Shift)	No of Participants (%) (Night Shift)	*p* Value
Fruit consumption	never	8 (1.9)	105 (25.1)	0.001
	rarely	41 (9.8)	185 (44.3)	
	sometimes	81 (19.4)	55 (13.2)	
	often	232 (55.5)	73 (17.5)	
	always	56 (13.4)	0 (0)	
Vegetable consumption	never	0 (0)	210 (50.2)	0.001
	rarely	35 (8.4)	137 (32.8)	
	sometimes	59 (14.1)	40 (9.6)	
	often	197 (47.1)	31 (7.4)	
	always	127 (30.4)	0 (0)	
Dairy consumption	never	0 (0)	135 (32.3)	0.001
	rarely	40 (9.6)	152 (36.4)	
	sometimes	87 (20.8)	100 (23.9)	
	often	235 (56.2)	31 (7.4)	
	always	56 (13.4)	0 (0)	
Legume consumption	never	0 (0)	120 (28.7)	0.001
	rarely	59 (14.1)	184 (44)	
	sometimes	227 (54.3)	90 (21.5)	
	often	128 (30.6)	8 (1.9)	
	always	4 (1)	16 (3.8)	
Poultry consumption	never	15 (3.6)	9 (2.1)	0.001
	rarely	43 (10.3)	27 (6.5)	
	sometimes	225 (53.8)	288 (68.5)	
	often	104 (24.9)	63 (15.1)	
	always	31 (7.1)	31 (7.4)	
Lunch meat consumption	never	16 (3.8)	37 (8.9)	0.001
	rarely	293 (70.1)	147 (35.2)	
	sometimes	89 (21.3)	189 (45.2)	
	often	20 (4.8)	45 (10.3)	
	always	0 (0)	0 (0)	
Red meat consumption	never	0 (0)	139 (33.3)	0.001
	rarely	178 (42.6)	78 (18.7)	
	sometimes	181 (43.3)	153 (36.6)	
	often	59 (14.1)	48 (11.5)	
	always	0 (0)	0 (0)	
Alcohol consumption	never	63 (15.1)	367 (87.8)	0.001
	rarely	158 (37.8)	47 (11.2)	
	sometimes	174 (41.6)	4 (1)	
	often	19 (4.3)	0 (0)	
	always	4 (1)	0 (0)	
Fish consumption	never	0 (0)	257 (61.5)	0.001
	rarely	73 (17.5)	148 (35.4)	
	sometimes	149 (35.6)	13 (3.1)	
	often	152 (36.4)	0 (0)	
	always	44 (10)	0 (0)	
Sweets/ice cream consumption	never	82 (19.6)	43 (10.3)	0.001
	rarely	236 (56.5)	241 (57.7)	
	sometimes	50 (12)	116 (27.8)	
	often	39 (9.3)	3 (1)	
	always	11 (2.6)	15 (3.4)	

**Table 3:** Relation between adherence to the Mediterranean diet and weight.

	Following Mediterranean Diet (Rarely) (*n* = 103)	Following Mediterranean Diet (Often) (*n* = 164)	Following Mediterranean Diet (Always) (*n* = 151)	*p* Value
Weight (kg) (mean ± SD)	90.8 ± 21.4	79.3 ± 5	74.5 ± 15	0.010

SD, standard deviation.

**Table 4:** Relationship between self-assessed nutritional habits and weight of the participants.

	Personal Beliefs about Eating Habits (Unhealthy) (*n* = 65)	Personal Beliefs about Eating Habits (Moderately Healthy) (*n* = 151)	Personal Beliefs about Eating Habits (Healthy) (*n* = 14)	Personal Beliefs about Eating Habits (Very Healthy) (*n* = 188)	*p* Value
Weight (kg) (mean ± SD)	91.6 ± 24	81.3 ± 10	78.4 ± 3	75.9 ± 14.1	0.015

SD, standard deviation.

## Data Availability

The data generated and analysed for this study can be found within the article and from the corresponding author on request.
